# Giant Mature Cystic Perihepatic Teratoma in an Adult Woman

**DOI:** 10.14309/crj.0000000000002286

**Published:** 2026-08-20

**Authors:** Bisher Sawaf, Nwal Hadaki, Mohammed Abu-Rumaileh, Akram Alnounou, Ajay Katwala, Sejal Gunaratnam, Umer A. Bhatti, Mohammad Al-Haddad

**Affiliations:** 1 Department of Internal Medicine, University of Toledo Medical Center, Toledo, OH; 2 Division of Gastroenterology and Hepatology, Indiana University School of Medicine, Indianapolis, IN; 3 Department of Internal Medicine, Homer Stryker MD School of Medicine, Western Michigan University, Kalamazoo, MI; 4 Indiana University School of Medicine, Carmel, IN

**Keywords:** germ cell tumors, teratomas, liver, extragonadal tumors, perihepatic tumors

## Abstract

Perihepatic teratomas are exceedingly rare extragonadal germ cell tumors that may mimic more common hepatic cystic lesions on imaging. We report a 38-year-old woman with several months of abdominal bloating and early satiety. Laboratory studies and tumor markers (α-fetoprotein and carcinoembryonic antigen) were unremarkable. Magnetic resonance imaging demonstrated a 17.7 cm complex cystic lesion adjacent to the left hepatic lobe with fatty components and a mural nodule. Elective laparotomy revealed a well-encapsulated mass arising from the lesser omentum, excised intact. Histopathology confirmed a mature cystic teratoma with elements from all 3 germ layers. The postoperative course was uneventful.

## INTRODUCTION

Teratomas are germ cell tumors composed of tissues derived from more than one embryonic germ layer, including ectoderm, mesoderm, and endoderm.^1,2^ They are classified as mature (benign, well-differentiated) or immature (with malignant potential).^3^ Although most arise in the gonads, extragonadal teratomas may develop along the body's midline, thought to result from aberrant migration of pluripotent germ cells during embryogenesis.^3,4^

Teratomas involving the liver account for less than 1% of reported cases, and perihepatic teratomas, those arising adjacent to, but not within, hepatic parenchyma, are rarer still.^5^ Their imaging features overlap with those of hydatid cysts and biliary cystadenomas, posing a diagnostic challenge. We report a large mature cystic perihepatic teratoma in an adult woman to highlight this rare entity in the differential diagnosis of complex abdominal masses.

## CASE REPORT

A 38-year-old woman presented to the clinic with several months of intermittent abdominal bloating and early satiety, worsening after meals. She denied fever, gastrointestinal bleeding, jaundice, or weight loss. Her history was notable for irregular menses, prior cesarean section, and cholecystectomy. She reported occasional alcohol and intermittent tetrahydrocannabinol (THC) gummy use. Family history was unremarkable.

Examination revealed epigastric and right upper quadrant tenderness without hepatomegaly or jaundice. Liver function tests, carcinoembryonic antigen ([CEA], 0.7 ng/mL), and α-fetoprotein were within normal limits with a slightly elevated carbohydrate antigen (CA) 19-9 level (Table 1). *Echinococcus* IgG, tuberculosis (TB) QuantiFERON, and stool studies were negative. A complete blood count showed mild leukocytosis.

**Table 1.** Basic work-up on presentation

Laboratory test	Measurement	Normal range
ALT	18 U/L	7–56 U/L
AST	22 U/L	8–48 U/L
ALP	94 U/L	44–147 U/L
Bilirubin	0.4 mg/dL	0.1–1.2 mg/dL
Albumin	4.2 g/dL	3.5–5.5 g/dL
CEA	0.7 ng/mL	0.0–3.0 ng/mL
AFP	2.2 ng/mL	<10 ng/mL
CA 19-9	39 U/mL	0–37 U/mL

AFP, α-fetoprotein; ALP, alkaline phosphatase; ALT, alanine transaminase; AST, aspartate transaminase; CA 19-9, carbohydrate antigen 19-9; CEA, carcinoembryonic antigen.

**Figure 1.**
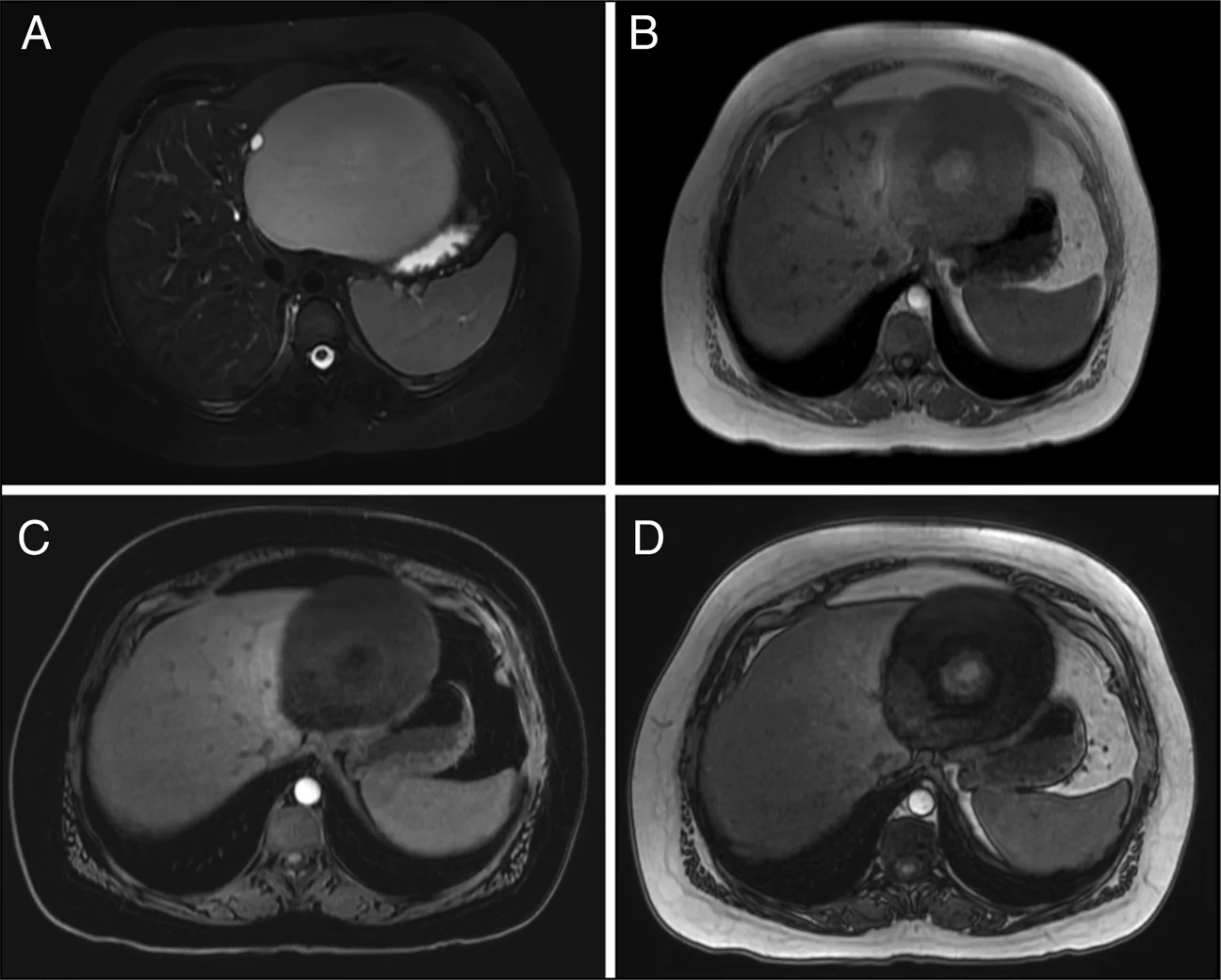
A 17.7 cm complicated cyst centered in the anterior upper abdomen alongside the left lobe of the liver. There is a smaller 11 mm mural cyst along its right superolateral wall (A), and in the nondependent upper portion, there is a 4.6 cm nodule (B–D). (A) Axial T2-weighted fat-suppressed MRI (navigation sequence). (B) Axial T1-weighted in-phase 3D LAVA precontrast breath-hold MRI. (C) Axial T1-weighted out-of-phase 3D LAVA precontrast breath-hold MRI. (D) Axial T1-weighted water-only 3D LAVA precontrast breath-hold MRI. MRI, magnetic resonance imaging.

Abdominal ultrasound demonstrated a large cystic lesion near the liver. Magnetic resonance imaging (MRI) showed a well-defined complex lesion adjacent to the lateral segment of the left hepatic lobe, measuring 17.7 × 11.6 × 15.8 cm, with intermediate T1/T2 signal, restricted diffusion, out-of-phase signal dropout (suggestive of fat), and a nonenhancing 3.6 cm central focus (Figure 1). Differential diagnoses included complicated hepatic cyst, biliary cystadenoma, teratoma, and, most compellingly, a hydatid cyst. Owing to the high suspicion of a hydatid cyst and possibility of leakage and infection spread, endoscopic ultrasound-fine-needle aspiration or fine-needle biopsy was not considered.

**Figure 2.**
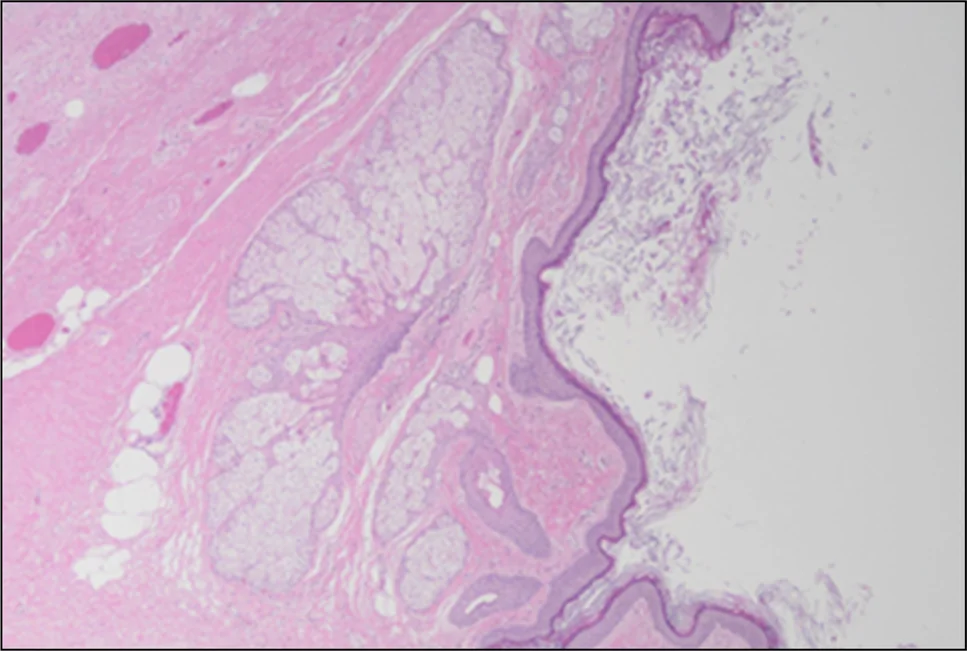
Hematoxylin and eosin stain (original magnification ×100) demonstrating abundant keratinous debris lined by mature squamous epithelium with sebaceous glands, consistent with elements derived from ectodermal differentiation.

**Figure 3.**
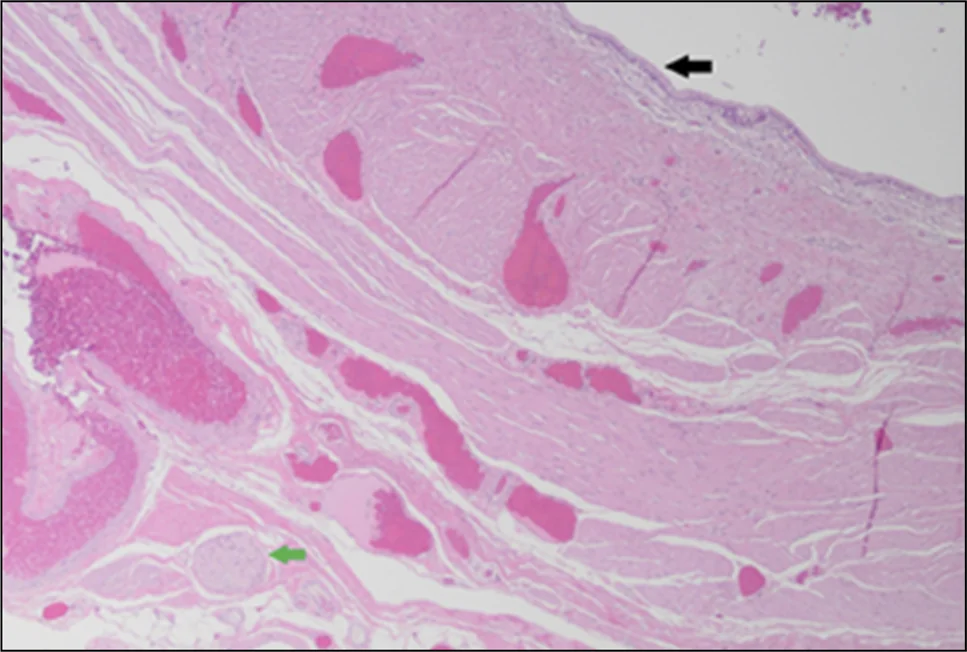
Hematoxylin and eosin stain (original magnification ×100) demonstrating mucinous epithelium resembling endocervical-type epithelium (black arrow) and a peripheral nerve bundle (green arrow), illustrating the presence of mature tissue derivatives.

Elective exploratory laparotomy revealed a well-encapsulated mass arising from the lesser omentum, abutting but not invading the liver or ovaries. The cyst was excised intact. Grossly, it measured 12.5 × 11.5 × 6.8 cm (559 g) and contained yellow-tan caseous material and hair, with a secondary intramural gelatinous cavity. Histopathology demonstrated mature squamous epithelium with sebaceous glands and hair follicles (ectoderm; Figure 2), mucinous endocervical-type epithelium (endoderm), and peripheral nerve bundles (mesoderm; Figure 3), confirming mature cystic teratoma. The patient recovered uneventfully, and follow-up MRI at 1 year showed no recurrence.

## DISCUSSION

Teratomas are composed of tissues derived from at least 2 of the 3 germ layers: ectoderm, mesoderm, and endoderm, which occur in both children and adults.^5^ Although most arise in the gonads, extragonadal teratomas can develop along the body's midline, including the mediastinum, retroperitoneum, sacrococcygeal region, and, rarely, near the liver.^6,7^ Teratomas involving the liver or surrounding tissues comprise less than 1% of all reported cases.^8,9^ Perihepatic teratomas develop adjacent to, rather than within, hepatic parenchyma; in our case, the mass arose from the lesser omentum, abutting but not invading the liver.

Several mechanisms have been proposed to explain these unusual locations. The most widely accepted theory suggests misplacement of pluripotent germ cells during embryologic migration, while alternative hypotheses include development from ectopic or autoamputated gonadal tissue.^10^ Regardless of origin, these lesions are often detected incidentally or when they grow large enough to cause mass-effect symptoms such as bloating and early satiety, as in our patient.^1,6^

Imaging plays a central role in initial evaluation, though preoperative diagnosis remains challenging because of the rarity of these tumors and their overlap with other cystic lesions. Ultrasonography typically demonstrates a well-circumscribed heterogeneous mass with mixed echogenicity reflecting variable fat, fluid, and soft-tissue content.^11^ Computed tomography (CT) is particularly useful for identifying macroscopic fat, calcifications, and fat-fluid levels.^12^ MRI further improves tissue characterization through high T1-weighted signal from sebaceous or fatty components, with chemical shift artifact, dependent layering, and Rokitansky protuberances as characteristic features; fat-suppressed sequences help distinguish teratomas from hemorrhagic lesions. Irregular wall thickening or solid components corresponding to immature neural tissue may raise concern for immature or malignant transformation.^11,12^ Despite these features, hepatic-region teratomas frequently mimic hydatid cysts or biliary cystadenomas, and definitive diagnosis typically requires histopathologic evaluation after resection.^13,14^ In our case, pathology confirmed a mature cystic teratoma containing elements from all 3 germ layers, underscoring the importance of considering this rare entity when evaluating large complex abdominal cystic lesions with inconclusive imaging.^7^

Most perihepatic teratomas are benign and cured by complete excision. However, approximately 2%–3% undergo malignant transformation, most often in younger patients or tumors containing immature elements.^15^ Squamous cell carcinoma is the most frequently reported malignancy, with adenocarcinoma and sarcoma also described.^15^

Compared with previously reported hepatic teratomas (Table 2), our case shared features common to benign adult presentations, including a large well-encapsulated mature cystic lesion, trilaminar histology, normal tumor markers, nonspecific mass-effect symptoms, and uneventful recovery after complete excision. Unlike most reports, which describe intrahepatic or hepatoduodenal ligament lesions, this tumor arose from the lesser omentum, near but not originating from hepatic parenchyma. It was also larger than most benign intrahepatic examples and radiologically mimicked other cystic hepatic lesions. In contrast to pediatric and immature teratoma cases, there was no malignant component and no need for adjuvant therapy, expanding the spectrum of hepatic-region teratomas to include rare, benign, extrinsic lesions with favorable outcomes after resection.

**Table 2.** Summary of key case reports highlighting hepatic teratoma findings

Author/yr	Patient demographics	Comorbidities	Clinical presentation	Primary tumor location	Metastasis location	Imaging findings	EGD/EUS/ERCP findings	Histological/biochemical findings	Treatment/management	Hospital course	Outcomes	Unique findings/comments
Winter and Freeny^16^, 1993	61 F	Multiple medical problems (unspecified); followed at university hospital for 20 yr	Incidental finding; vague calcific densities in the RUQ first noted on plain abdominal films ∼20 yr prior (age 44); interval growth and tooth-like appearance became apparent over subsequent decade; presented acutely with upper gastrointestinal bleeding	Right lobe of liver (intrahepatic)		Serial plain abdominal films (1974–1991): Interval development and growth of calcific densities in right upper quadrant with tooth-like appearance. CT (1991): Predominantly cystic intrahepatic mass containing teeth (HU = 17), confirming odontogenic origin of calcifications		Radiologic diagnosis of teratoma based on odontogenic calcifications within cystic hepatic mass; considered pathognomonic	Conservative; no surgical intervention performed due to patient's multiple medical comorbidities	Not reported	Not reported	Presence of tooth within a cystic hepatic lesion considered pathognomonic for teratoma; only tumor type known to produce odontogenic calcification in the liver
Sasaki et al^17^, 2005	38 M		Fever, right hypochondrial mass	Hepatoduodenal ligament		CT: Cystic structure with fat-fluid level and calcification; barium meal: tooth-like calcification	ERCP: Bile duct compression	Mature cystic teratoma; CA19-9 elevated; minor LFT changes	Tumor excision, cholecystectomy, Roux-en-Y choledochojejunostomy	Uncomplicated recovery	No recurrence	Rare hepatoduodenal ligament teratoma
Rahmat et al^7^, 2006	46 M		Progressive jaundice, fever, chills, rigors, weight loss, pale stools, pruritus, vomiting, upper abdominal pain	Left lobe of liver		US: Heterogeneous solid mass; CT: fat, calcification, soft tissue; MRCP: Fat + soft tissue mass compressing CBD		Mature benign teratoma; bilirubin total 99 mmol/L, conjugated 93 mmol/L; ALT 127 IU/L; AST 50 IU/L; ALP 196 IU/L; GGT 124 IU/L; hepatitis serology negative	Exploratory laparotomy, mass excision with atrophic left lobe resection	Postoperative recovery uneventful	Discharged; follow-up ongoing	Mass compressing CBD without direct communication to biliary tree
Moll et al,^14^ 2009	3 M		Hard RUQ mass; elevated transaminases	Right lobe of liver (intra-/extra-hepatic)		MRI: Large heterogeneous mass with cystic and solid components; heterogeneous enhancement; partial IVC compression; enlarged after chemotherapy		Mixed hepatoblastoma and malignant teratoma; embryonal/fetal areas with neuroblastomatous and rhabdomyosarcomatous differentiation; AFP, NSE elevated; HepPar-1+, synaptophysin+, desmin+; MYCN negative	PEI chemotherapy → extended right hemihepatectomy → adjuvant IPA chemotherapy	Uncomplicated	No recurrence at 3 yr	Second reported case; chemotherapy effective in hepatoblastoma component only
Gupta et al^6^, 2013	4 M		1-yr history of constipation, loss of appetite, mild abdominal distension, and intermittent fever; firm, nontender hepatomegaly extending to right lumbar region	Caudate lobe of liver		US: Multiloculated round cystic lesion in caudate lobe (4.3 × 4.5 × 4 cm) with internal septations; no calcification or fat on US. CT: Well-defined contrast-enhancing multiloculated cystic lesion (43.8 × 43 × 41 mm) with multiple septations and calcification in wall; no fat component identified	Not performed; hydatid serology (IgG ELISA) negative	Mature cystic teratoma; elements included skin, bone, glial tissue, tooth enamel and pulp, lymphoid aggregates, and choroid plexus; no immature component. Postoperative AFP 0.82 ng/mL, β-hCG 1.2 mIU/L — both normal; Hgb 10.5 g/dL (anemia); LFTs within normal limits	Laparotomy with partial excision and marsupialization of cystic lesion		Doing well at 1-yr follow-up; no residual mass or recurrence on follow-up US	Absence of fat on both US and CT made radiologic diagnosis difficult; marsupialization rather than complete excision raises risk of recurrence; caudate lobe involvement is uncommon
Krainev et al^18^, 2018	33 F		Chronic nonlocalizable abdominal pain	Liver segments 7 and 8		CT: Dome of liver mass; MRI: Complex lesion with cystic, fat, calcification	Upper and lower endoscopy: Negative	Benign cystic teratoma with carcinoid tumor	Laparoscopic partial hepatectomy	Uneventful	Negative margins, stable	Coexisting carcinoid tumor within teratoma
Ravikumar et al^19^, 2018	7-day-old M		Antenatal abdominal mass, distension	Hepatoduodenal ligament		MRI/US: Large abdominal mass		Immature teratoma grade III; AFP 20,000; B-HCG 1.2	Excision with repair of portal triad	Required massive transfusion; recovered	Normal LFTs at 6 months	Portal vein, hepatic artery, CBD injury repaired successfully
Davila-Ruiz et al^20^, 2020	39 F	Right ovarian cyst resection in 2016	RUQ mass, pain, weight loss, fever, asthenia	Suprahepatic region		CT: Heterogeneous solid/cystic mass compressing liver, gastric cavity, abdominal viscera; portal system compression		Mixed germ cell tumor: Mature cystic teratoma (70%) + yolk sac tumor (30%); AFP 3622; CA125 485; CA 19-9 normal; CEA normal	Surgical resection	Uncomplicated	No recurrence at 1 yr	Rare suprahepatic location
Kovalenko et al^5^, 2021	52 F	History of mature ovarian teratoma 10 yr earlier	Incidental solid mass in left liver lobe, initially suspected cholangiocarcinoma	Segment I liver		CT/MRI: Heterogeneous lesion with calcifications		Benign mature teratoma with ectopic thyroid tissue	Segment I resection		No recurrence reported	Ectopic thyroid tissue within hepatic teratoma
Saba et al^13^, 2023	27 M		RUQ pain for 5 months	Left lobe of liver		CT: Encapsulated extrahepatic/subhepatic lesion with fat, septations, calcification		Mature cystic teratoma; AFP insignificant; LFTs normal	Segmental liver resection + cholecystectomy	Uncomplicated	No recurrence reported	

AFP, α-fetoprotein; ALP, alkaline phosphatase; ALT, alanine transaminase; AST, aspartate transaminase; B-HCG, β-human chorionic gonadotropin; CA 19-9, carbohydrate antigen 19-9; CBD, common bile duct; CEA, carcinoembryonic antigen; CT, computed tomography; EGD, esophagogastroduodenoscopy; IgG ELISA, immunoglobulin G enzyme-linked immunosorbent assay; ERCP, endoscopic retrograde cholangiopancreatography; EUS, endoscopic ultrasound; GGT, γ-glutamyl transferase; HU, Hounsfield Units; LFTs, liver function tests; MRCP, magnetic resonance cholangiopancreatography; MRI, magnetic resonance imaging; MYCN, v-myc avian myelocytomatosis; NSE, neuron-specific enolase; RUQ, right upper quadrant.

Perihepatic teratomas are exceedingly rare extragonadal germ cell tumors that may closely mimic more common hepatic and perihepatic cystic lesions on imaging. This case illustrates a giant mature cystic teratoma arising from the lesser omentum, emphasizing the importance of including this rare entity in the differential diagnosis of large complex abdominal cystic masses. Complete surgical excision remains both diagnostic and curative, with excellent outcomes in benign cases.

## DISCLOSURES

Author contributions: B. Sawaf: Conceptualization, literature review, manuscript drafting (case presentation and discussion), and manuscript revision. N. Hadaki: Literature review, manuscript drafting (introduction and discussion), and figure preparation. M. Abu-Rumaileh: Clinical data collection, manuscript drafting (case presentation), and critical revision. A. Alnounou: Literature review, table preparation, and manuscript editing. A. Katwala: Clinical data collection, interpretation of imaging findings, and manuscript review. S. Gunaratnam: Literature review, manuscript editing, and proofreading. UA Bhatt: Critical revision of the manuscript for important intellectual content and supervision. M. Al-Haddad: Conceptualization, supervision, critical revision of the manuscript, and final approval of the version to be submitted. All authors have read and approved the final version of the manuscript and agree to be accountable for all aspects of the work. M. Al-Haddad is the article guarantor.

Financial disclosure: None to report.

Informed consent was obtained for this case report.

## ABBREVIATIONS:

CEA: carcinoembryonic antigen
CT: computed tomography
MRI: magnetic resonance imaging
